# Novel nanosensor of cobalt(II) and copper(II) constructed from graphene quantum dots modified with Eriochrome Black T

**DOI:** 10.1038/s41598-022-17616-y

**Published:** 2022-08-01

**Authors:** Leila Vahab, Sajjad Keshipour

**Affiliations:** Nanotechnology Department, Faculty of Science, Urmia University, Urmia, Iran

**Keywords:** Pollution remediation, Sensors

## Abstract

Detection of heavy transition metals is of importance protecting humans and the environment from their toxicity. Amongst them, copper(II) and cobalt(II) need more attention due to their wide applications in industry, in which exposures with excess amounts of them cause heathy concerns. Hence, fast and efficient distinguishing copper(II) and cobalt(II) could be worthy. While electrochemical approaches could determine these cations, expensive instrumentation and time-consuming analysis are significant drawbacks, leading to concentrations on spectroscopic and colorimetric pathways. Herein, graphene quantum dot was modified with Eriochrome Black T (EBT) to generate a novel sensor with the ability of sensing mentioned cations. This new nanocompound demonstrated new optical properties when complexed with cobalt(II) in natural pH, and copper(II) in acidic media. Both cations successfully were detected by the new nanosensor through revealing distinct spectroscopic signals. Moreover, cobalt(II) was distinguished by this sensor colorimetrical, appearing green solution. Linear ranges of cobalt(II) and copper(II) were obtained as 0.02–0.08 M and 0.015–0.2 M, with the limit of detections 0.014 and 0.018 M, respectively.

## Introduction

According to the classical definition, heavy metals are classified as elements with molecular weights ranging from 63.5 to 200.6, which most of them like Cu, Co, Pd, Hg, and Cd have potential to threat the environment and mankind^1^. Some of these elements such as Co and Cu are widely employing in the industry, which their toxicity could be significant concerns for the human^2^. Alarms and asthma, cardiac and thyroid damage, heart failure and heart disease, and elevated red blood cells are some disorders caused by exposure with an excess amounts of Co(II). Hereditary copper metabolism disorders and neurodegenerative ailments are the issues ascribed to Cu(II) pollutions^3^. These concerns persuade researchers to continue seeking for efficient sensors to swift detect of the trace amounts of toxic metals especially in industrial wastes as the primary source of the pollution^4^. Developing well-functioning, cost-effective, rapid, and recoverable sensors could utterly refute traditional detection methods like Atomic Absorption Spectroscopy (AAS), Induced Coupled Plasma, Ion Chromatography, and electrochemistry^5^. Optical sensors have created fantastic progress in the metal distinguishing, in which all the positives are accessible. A wide variety of synthetic materials have deserved optical metal determination by either spectroscopic or colorimetric approaches^6^. It was demonstrated that graphene-based sensors productively detect heavy metals due to the superior optical activity, arising from a large conjugated π-network^7,8^. CoS/reduced porous graphene oxide for colorimetric detection of Hg(II)^9^, CuS and NiS nanoparticles-decorated on porous-reduced graphene oxide for the colorimetric detection of Hg(II)^10^, and AuNPs@MoS_2_/reduced graphene oxide immunosensor of the electrochemical neuron-specific enolase (NSE)^11^ are some examples of an unaccountable number of reports on graphene sensors for the detection of organic and mineral compounds. Moreover, graphene quantum dot (GQD) applications are boasting due to its high electrical conductivity, chemical stability, and surface area^12^. A remarkable number of GQD composites were employed in the synthesis of sensors such as serine and histidine-functionalized GQD for detecting carbendazim^13^, gold-histidine functionalized GQD-graphene hybrid for sensing chlorpyrifos^14^, GQD/multi-walled carbon nanotubes for ascertaining Interleukin-6^15^, gold nanoparticles functionalized sulfur-doped GQD and h-ZnS-CdS NC for determining Interleukin-6^16^, and nitrogen and boron-doped GQD for distinguishing cardiac troponin I^17^.

**Scheme 1:**
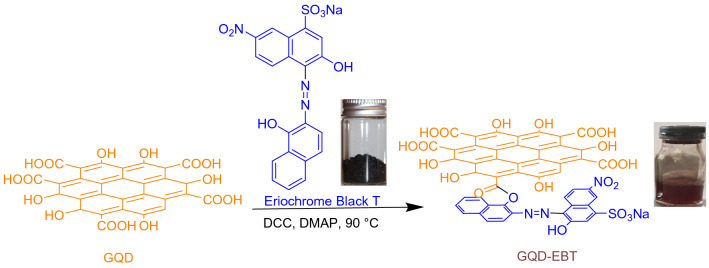
Synthesis of GQD-EBT.

Eriochrome Black T (EBT) is an azo dye with an appropriate chemical structure to be employed as a complexometric indicator of Ca(II). While the indicator plays a crucial role in determining water hardness, pollution created by this azo dye causes severe issues for plants and animals. This concern has led to a vast number of reports on the EBT removal from water wastes, and encouraged scientists to come up with the idea of binding EBT on a support^18^. Surprisingly, EBT deposited on various supports demonstrated novel application in detecting various organic and inorganic materials. For example, poly(benzopyrene) films doped with EBT as a Pb^2+^-sensitive sensor^19^, poly(EBT)-modified carbon paste an electrode for Hg^2+^ distinguishing^20^, EBT/graphite composite for Theophylline determination^21^, and poly(EBT) on glassy carbon electrode for recognition of Isoniazid are some successful reports in this area^22^. Most of these detections were focused on electrochemical pathways, while we believe that EBT has significant optical potential on account of its extensive π-system. We recently demonstrated that the perching of Alizarine Red S on GQD generates a powerful sensor for detecting metal cations^23^. Easy, rapid, and cost-effective synthesis of GQD in combination with sustainability, and facile modification are some characteristics of this material that encouraged us to its modification with EBT^24–26^. Therefore, GQD was modified with EBT (GQD-EBT) to obtain a novel sensor of metal cations via esterification reaction (Scheme 1). This reaction efficiently was progressed by the *N*,*N*′-dicyclohexylcarbodiimide (DCC) and 4,4′-dimethylaminopyridine (DMAP) as the esterification agents^27,28^. The sensor successfully detected two significant metal cations, including Co^2+^ and Cu^2+^, which their fast detections are not well-stablished till now^29–31^.

## Experimental section

### Materials and methods

All reagents were purchased from Sigma-Aldrich and used without further purification. TEM, and SEM micrographs were obtained by  FEI Tecnai G2 F20 SuperTwin TEM, and FESEM TESCAN MIRA3, respectively. FT-IR of AVATAR FTIR instrument of Thermo Company in the range of 600–4000 cm^−1^ was used to determine functional groups of compounds. XRD of XRD Philips PW1730 was employed with the Cu k_α_ (λ = 1.540598 nm), a voltage of 140 kV, and scanning from 10° to 80°. UV–Vis spectra were obtained by WPA Biowave LifeScience UV–Vis spectrometer using quartz cuvettes with a path length of 10 mm in H_2_O as the solvent.

### Synthesis of GQD-EBT

GQD (2.0 g), DCC (0.4 g), and DMAP (0.03 g) were added to a round bottom flask containing 9 ml of dimethyl sulfoxide, and 1 ml of H_2_O. The resulted mixture was stirred magnetically at 90 °C for 10 min. Then, EBT (0.5 g) was added to the reaction mixture, and stirring was continued for 2 h. Next, the precipitate was filtered off, and the residue was washed with H_2_O (3 × 10 ml) to give GQD-EBT as a dark solid after drying at 60 °C.

### General procedure for detecting M^x+^

To 5 ml of an adjusted M^x+^ solution (pH = 3 with HCl and pH = 10 with NaOH) in the desired concentration, 0.05 g of GQD-EBT was added. Color changes were recorded as the addition of EBT-GQD. UV–Vis spectroscopies were carried out after calibration of the instrument with the standard solution of media.

### Interfere study of cations on Co^2+^

GQD-EBT (0.05 g) was added to 10 ml of 0.02 M solution of Ca^2+^, Cu^2+^, Hg^2+^, Pb^2+^, Fe^3+^, Cd^2+^, Al^3+^, and Ni^2+^. Color changes were recorded as the addition of EBT-GQD. UV–Vis spectroscopy was carried out after calibration of the instrument with the standard media solution.

## Results and discussion

### Characterization of GQD-EBT

**Figure 1:**
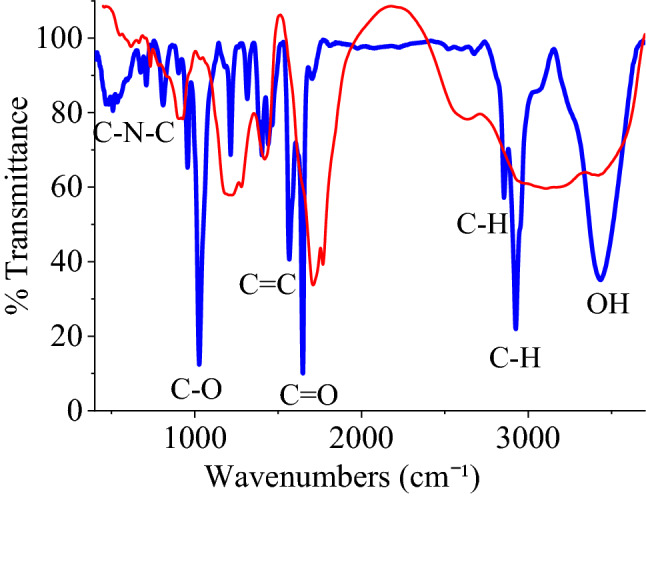
FT-IR spectra of GQD (red) and GQD-EBT (blue).

Organic transformations could be quickly approved by FT-IR spectroscopy, which highlights the addition, elimination, and transformation of functional groups with active vibrational modes. FT-IR spectra of GQD before and after modification indicated remarkable changes. Three prominent peaks of GQD appeared in both spectra, including stretching modes of OH, C=O, and C=C. The peak of C=O indicated a blueshift when GQD was modified with EBT due to the esterification reaction (Fig. 1)^24^.

**Figure 2:**
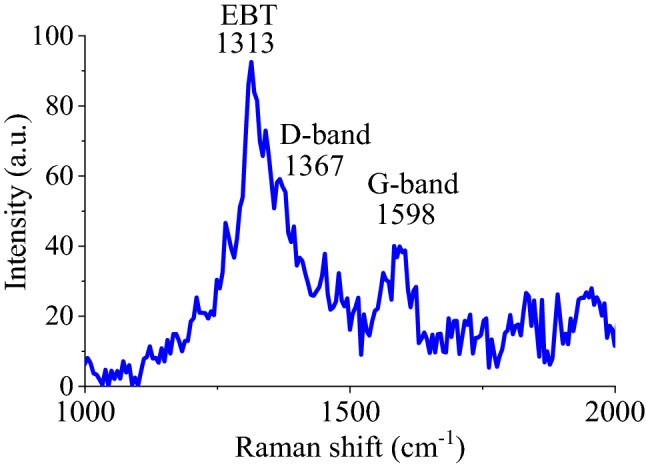
Raman spectrum of GQD-EBT.

Raman spectrum of GQD-EBT was also employed to characterize the structure with the ability to provide worthy information about the graphene-based structures. The spectrum of GQD-EBT demonstrated peaks of D-band at 1313 cm^−1^ attributed to the bending vibrations of C-H at the defects/edges and G-band at 1598 cm^−1^ ascribed to in-plane C=C vibrations, respectively. The high intensity of the D-band compared to G-band is a reliable sign for the presence of abundant functional groups on GQD (Fig. 2). Also, a strong peak appeared at 1313 cm^−1^ for EBT relating to C=C bonds^32^.

**Figure 3:**
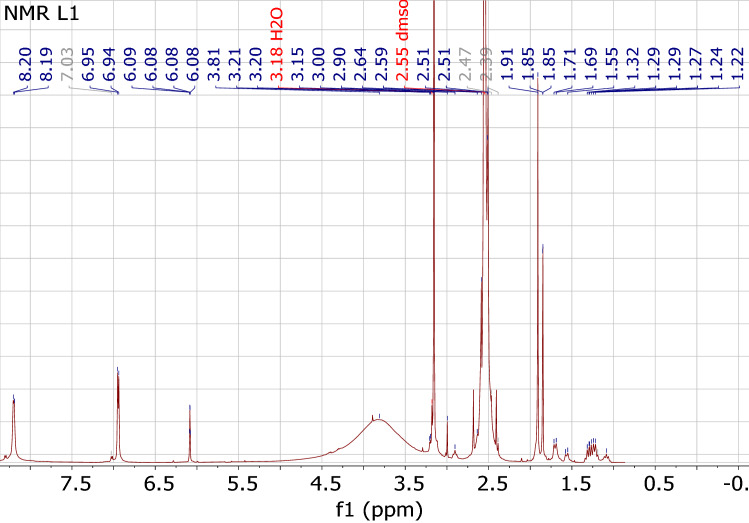
^1^H NMR spectrum of GQD-EBT.

^1^H NMR spectroscopy was also employed to affirm the modification reaction, where H-aromatic of EBT in the GQD-EBT spectrum could confirm loading of the indicator on GQD. Spectrum of GQD-EBT indicated peaks for H-aromatic at about 7.0 and 8.2 ppm (Fig. 3), which did not observe in the GQD spectrum (Figure S1). These peaks clearly confirm the loading of EBT on GQD.

**Figure 4:**
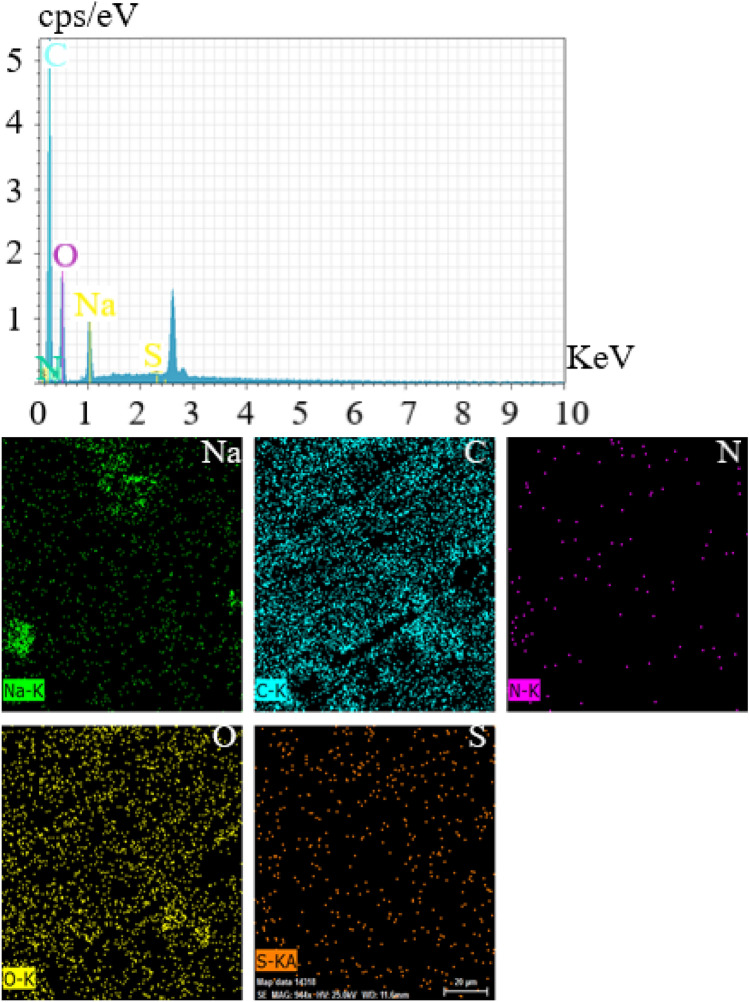
EDX and elemental mapping of GQD-EBT.

Elemental analysis was conducted by Energy Dispersive X-ray Spectroscopy (EDX) on GQD-EBT to determine various elements in the structure (Fig. 4). X-ray detector of SEM instrument revealed C and O atoms of GQD with high intensities. The analysis also indicated atoms ascribed to EBT, including S, Na, and N. To disclose the EBT distribution on GQD surface, elemental mapping was carried out, in which homogeneous distribution of S, N, and Na atoms observed (Fig. 3). This homogeneity in the elements’ dispersal implies success of the homogeneous sensor synthesis.

**Figure 5:**
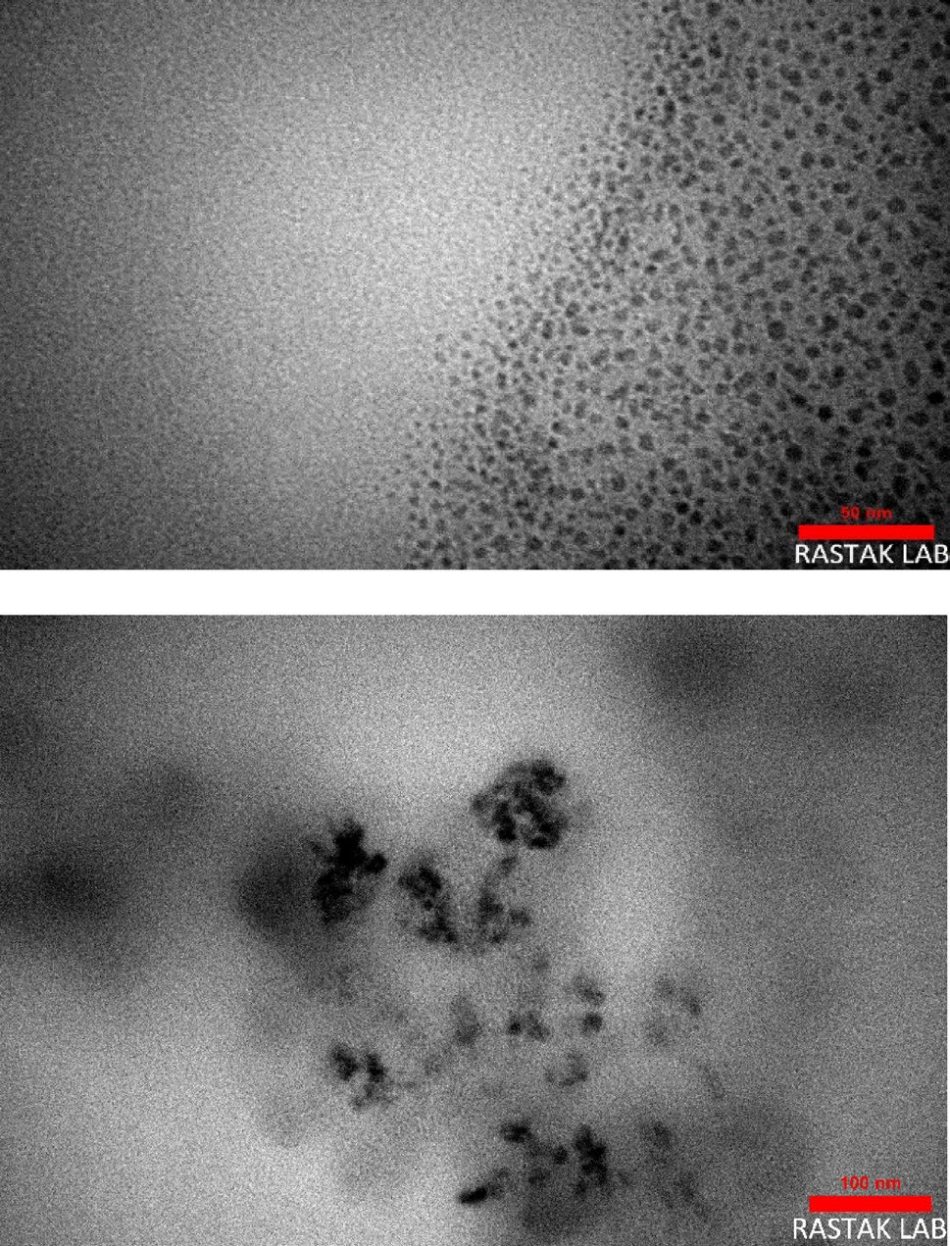
TEM micrographs of GQD and GQD-EBT.

TEM micrographs of GQD were prepared before and after modification to see the structure and changes made during EBT loading on GQD (Fig. 5). TEM image of GQD showed the formation of nanoparticles in the range of 4–8 nm. The reaction time of citric acid transformation to graphene derivatives determines the dimensions of the synthesized particles with the fine particles in a shorter time^33^. TEM of GQD-EBT demonstrated that the thermal treatment of GQD during the modification reaction led to the nanoparticles growing, although they are still GQD with a size under 100 nm.

### Detecting heavy metals by GQD-EBT

**Figure 6:**
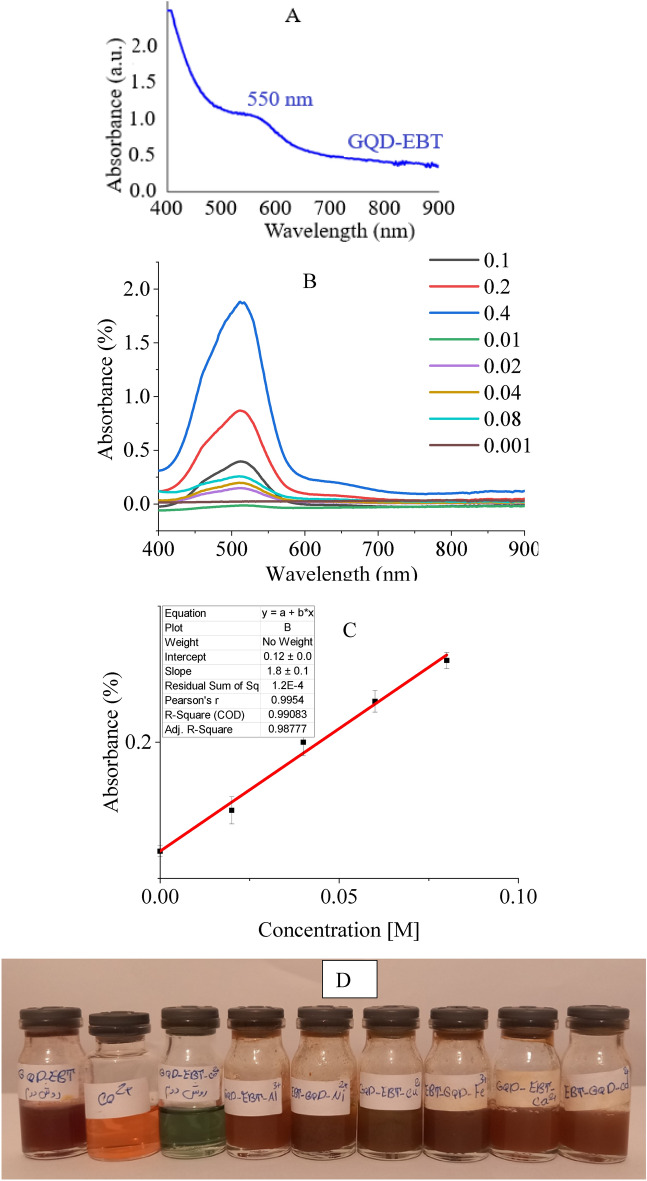
UV–Vis spectrum of GQD-EBT (**A**) and GQD-EBT-Co^2+^ in various concentrations of Co^2+^ at natural pH (**B**), a linear range of absorbance in various concentrations (**C**), and colorimetric detection of Co^2+^ (**D**).

The optical behavior of GQD-EBT was studied by a spectrophotometer in the presence and absence of metal cations. GQD-EBT showed an absorption at 550 nm without any cation in a natural pH (Fig. 6). While the addition of GQD-EBT to most of the cations solutions (0.02 M) such as Ca^2+^, Cu^2+^, Hg^2+^, Pb^2+^, Fe^3+^, Cd^2+^, Al^3+^, and Ni^2+^ did not create impressive changes in the absorption wavelength in the natural pH, charging to Co^2+^ solution (0.02 M) led to the redshift of absorption to 514 nm (Fig. 6). Surprisingly, GQD-EBT could not spectroscopically or colorimetrical detect Ca^2+^ opposite to EBT. Intriguingly, the new sensor distinguished Co^2+^ colorimetrical by changing the brown GQD-EBT solution to green. The amount of GQD-EBT was optimized as 0.05 g, in which the best color detection of Co^2+^ was obtained at the lowest amounts of the sensor. A standard addition technique for Co^2+^ detection by GQD-EBT revealed a calibration curve of y = 1.80x + 0.12 with a linear range lying between 0.02–0.08 M. Limit of detection (LOD) was calculated as 0.014 M.

**Figure 7:**
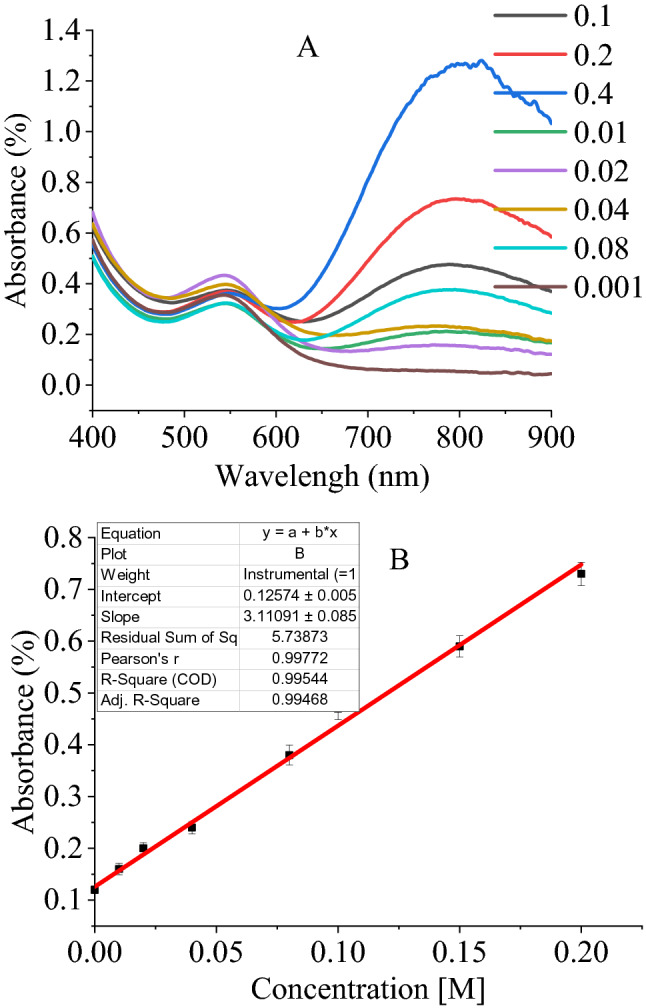
UV–Vis spectrum of GQD-EBT and GQD-EBT-Co^2+^ in various concentrations of Cu^2+^ at pH = 3 (**A**), and linear range of absorbance in various concentrations (**B**).

To elucidate the effect of solution pH on the detection ability of GQD-EBT, recognitions of the mentioned cations were carried out in two pHs of 10 and 3. As the first result, the detection of Co^2+^ was lost in both acidic and basic conditions. However, the result of this experiment was fascinating for Cu^2+^ in pH = 3, in which a new peak appeared at about 800 nm in addition to a peak of 550 nm (Fig. 7). A standard addition technique for Cu^2+^ detection by GQD-EBT demonstrated a calibration curve of y = 3.0653x + 0.1314 with a linear range lying between 0.015–0.2 M and an LOD of 0.018 M. The colorimetric distinguishing of Cu^2+^ was impossible due to the no detectable color change with the addition of the cation to the sensor solution.

Selectivity of the sensor was examined in a solution containing various cations at pH = 7, leading to the successful determination of Co^2+^ by spectroscopic and colorimetric methods. The peak of Co^2+^/GQD-EBT was observed in the spectrum of the solution which gained deep green color, indicating noninterference of other cations. Investigation of the selectivity at pH = 3 was also showed the possibility of the Cu^2+^ detection spectroscopically by observing the related peak at about 800 nm. Real samples were studied by GQD-EBT, with the various concentrations of Co^2+^ and Cu^2+^ in the tape and river water, in which both cations were successfully distinguished by the sensor after adjusting pH. Table 1 describes the results of detecting various concentrations of Co^2+^ and Cu^2+^.

**Table 1:** The results of Co^2+^ and Cu^2+^ detections by GQD-EBT in the real samples (N = 3).

Sample	Real Co^2+^ [M]	found Co^2+^ [M]	Recovery (%)	RSD (%)	Real Cu^2+^ [M]	found Cu^2+^ [M]	Recovery (%)	RSD (%)
Tape water	0.020	0.021	105	2.79	0.020	0.020	105	2.84
Tape water	0.050	0.051	102	2.0	0.100	0.103	103	1.7
Tape water	0.080	0.079	98.8	1.3	0.200	0.192	96	0.5
River water	0.020	0.019	95	3.1	0.020	0.019	95	1.0
River water	0.050	0.049	96.4	1.2	0.100	0.098	98.0	1.8
River water	0.080	0.078	96.2	1.3	0.200	0.195	97.5	1.1

A remarkable number of sensors has been reported for the Co^2+^ and Cu^2+^ distinguishing, in which some of them indicated superior sensitivity and selectivity than GQD-EBT^23,30,31^. However, introducing new approach to the functionalizing GQD with an indicator such as EBT could be a promising pathway to afford a lot of new sensors via this protocol. Easy synthesis of GQD from inexpensive citric acid, high optical activity of GQD, facile GQD modification through carboxylic acid groups, useful changing in the GQD’s optical properties due to modification with an indicator are some of significant features highlighted in this study. Therefore, synthesis of an economical sensor for the fast detection of Co^2+^ and Cu^2+^ are most valuable positives of this pathway compared to the most of reported sensors.

## Conclusion

In summary, a novel sensor was synthesized from GQD and EBT by the facile and efficient pathway of esterification reaction. GQD modified EBT demonstrated different performance in the detection of cations compared to EBT, such as inability in Ca^2+^ detection. The new sensor successfully detected Co^2+^, and Cu^2+^ in natural, and acidic solutions, respectively. Moreover, colorimetric detection of Co^2+^ was also affordable with a green color induced by the sensor. This study offers that modifying GQD with optically active compounds could generate new nanomaterials with characteristic optical activities. Therefore, a considerable number of researches could be conducted focusing on the synthesis of new sensors for the detection of heavy metal cations.

## Supplementary Information

Supplementary Information.
