# Supplementary material for: Novel nanosensor of cobalt(II) and copper(II) constructed from graphene quantum dots modified with Eriochrome Black T
Source: Sci Rep. 2022 Aug 1;12:13179. doi: 10.1038/s41598-022-17616-y (PMC9343609; doi:10.1038/s41598-022-17616-y)
Supplement: Supplementary file 1 — Supplementary Information. [file 41598_2022_17616_MOESM1_ESM.docx]

**Supporting Information:**

**Novel Nanosensor of Cobalt(II) and Copper(II) Constructed from Graphene Quantum Dots Modified with Eriochrome Black T**

Leila Vahab, Sajjad Keshipour^*^

Nanotechnology Department, Faculty of Science, Urmia university, Urmia, Iran; Email: S.keshipour@urmia.ac.ir


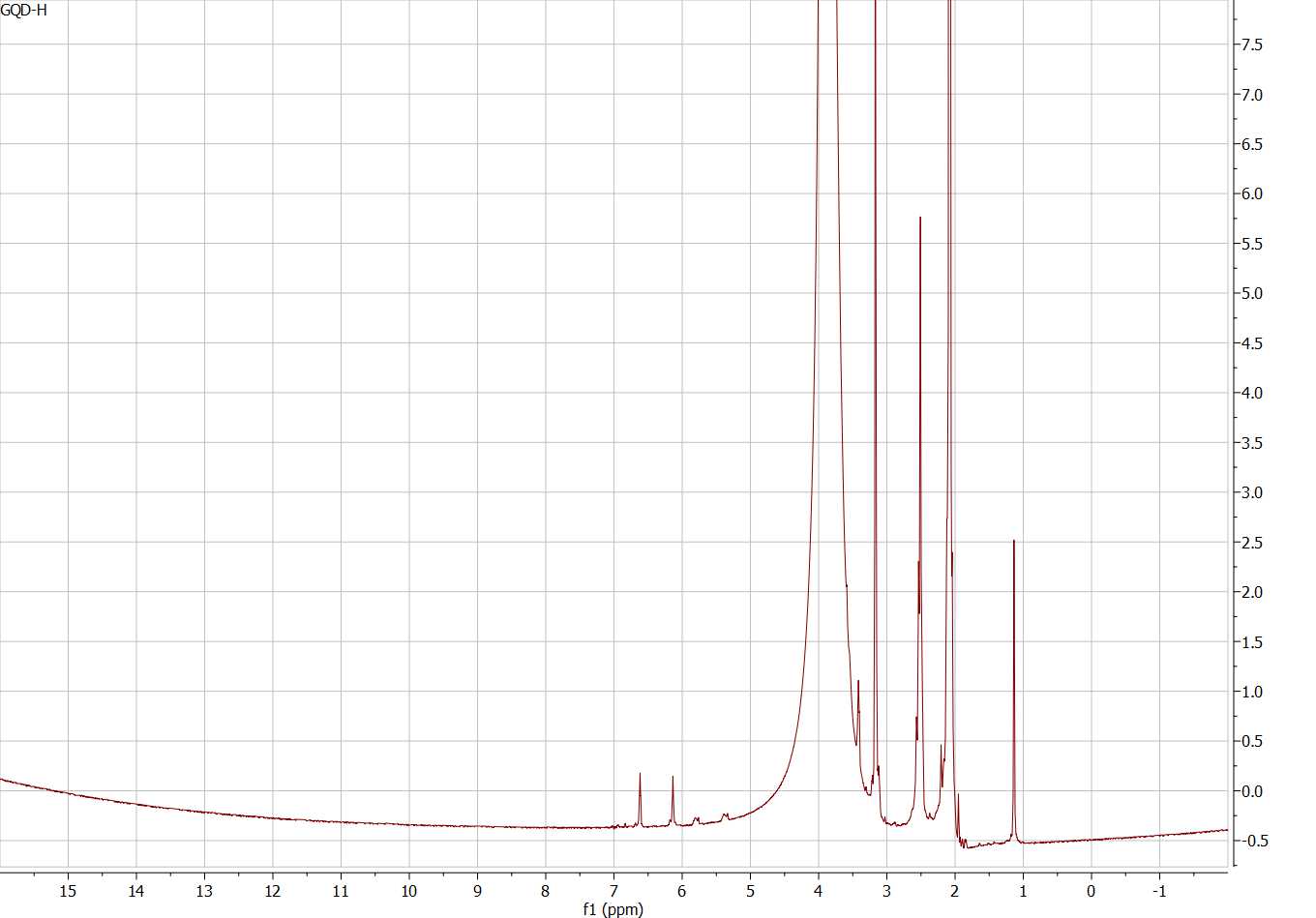


**Figure S1.** ^1^H NMR of GQD
